# Physiological Effects and Human Health Benefits of *Hibiscus sabdariffa*: A Review of Clinical Trials

**DOI:** 10.3390/ph15040464

**Published:** 2022-04-12

**Authors:** Efigenia Montalvo-González, Zuamí Villagrán, Sughey González-Torres, Laura Elena Iñiguez-Muñoz, Mario Alberto Isiordia-Espinoza, José Martín Ruvalcaba-Gómez, Ramón Ignacio Arteaga-Garibay, José Luis Acosta, Napoleón González-Silva, Luis Miguel Anaya-Esparza

**Affiliations:** 1 Integral Food Research Laboratory, National Technological of Mexico/Technological Institute of Tepic, Av. Tecnologico 2595, Tepic 63175, Mexico; emontalvo@ittepic.edu.mx; 2 Department of Health Sciences, Division of Biomedical Science, University Center of Los Altos, University of Guadalajara, Av. Rafael Casillas Aceves 1200, Guadalajara 47600, Mexico; blanca.villagran@academicos.udg.mx (Z.V.); sgonzalez@cualtos.udg.mx (S.G.-T.); 3 División of Natural and Technological Exact Sciences, Southern Region University Center, University of Guadalajara, Av. Enrique Arreola Silva 883, Guadalajara 49000, Mexico; laura.iniguez@academicos.udg.mx; 4 Department of Clinics, Division of Biomedical Sciences, Institute of Research in Medical Sciences, Los Altos University Center, University of Guadalajara, Av. Rafael Casillas Aceves 1200, Guadalajara 47600, Mexico; mario.isiordia@academicos.udg.mx; 5 National Center for Genetic Resources, National Institute of Forestry, Agriculture and Livestock Research, Boulevard de la Biodiversidad 400, Tepatitlan de Morelos 47600, Mexico; ruvalcaba.josemartin@inifap.gob.mx (J.M.R.-G.); arteaga.ramon@inifap.gob.mx (R.I.A.-G.); 6 Interdisciplinary Research Centre for Integral Regional Development Sinaloa Unit, National Polytechnic Institute, Boulevard Juan de Dios Bátiz 250, Guasave 81049, Mexico; jlacostar@ipn.mx; 7 Department of Livestock and Agricultural Sciences, University Center of Los Altos, University of Guadalajara, Av. Rafael Casillas Aceves 1200, Guadalajara 47600, Mexico

**Keywords:** *Hibiscus sabdariffa*, calyx, clinical trials, health benefits, bioactive compounds

## Abstract

*Hibiscus sabdariffa* Linn. Malvaceae (HS) is characterized by its edible calyxes. The HS calyxes are widely used for cosmetic, food, and medicinal applications. According to ethnobotanical evidence, decoction, infusion, or maceration extracts from HS calyxes have been used in folk medicine to treat many ailments. Moreover, several in vitro and in vivo studies have demonstrated the pharmacological properties and potential human health benefits of HS consumption. On the other hand, the evaluation of the physiological effects and health benefits of HS in clinical studies is most challenging. Therefore, this narrative review summarizes and discusses the physiological effects and health benefits of HS calyxes reported in clinical trials. Preparations obtained from HS calyxes (extracts, infusions, decoction, teas, beverages, capsules, and pills) are used as non-pharmacological therapies to prevent/control diverse chronic non-communicable diseases. The most-reported HS health benefits are its antihypertensive, antidyslipidemic, hypoglycemic, body fat mass reduction, nephroprotective, antianemic, antioxidant, anti-inflammatory, and anti-xerostomic activities; these effects are associated with the phytochemicals found in HS. Moreover, no adverse effects were reported during the clinical trials. However, clinical studies exhibited some limitations; thus, further studies are required to validate the clinical efficacy of HS in large-scale studies with higher doses and a good experimental design

## 1. Introduction

According to the World Health Organization, chronic non-communicable diseases (NCDs) are the principal cause of death globally and refer to a group of metabolic disorders including hyperglycemia, hyperlipidemia, hypercholesterolemia, diabetes mellitus type 2, hypertension, arteriosclerosis, cancer, and chronic lung illnesses. NCDs affect people of all ages, especially in middle- and low-income countries [1]. Recently, it has been reported that 71% of all deaths in the world are associated with NCDs, creating severe socioeconomic and medical problems [2]. In this context, the prevention and control of NCDs may be due to reducing risk factors associated with these diseases such as raised blood pressure, overweight, obesity, high glucose, and lipid blood levels by consumption of functional foods, including herbal preparations, particularly *Hibiscus sabdariffa* Linn. Malvaceae (HS) [3].

HS is commonly known as roselle, jamaica, red sorrel, Indian sorrel, wonjo, and karkade, and it is native to India and Malaysia [4]. The plant belongs to the Malvaceae family and is widely distributed and cultivated worldwide in tropical and subtropical regions, including China, Thailand, Indonesia, Egypt, Sudan, Saudi Arabia, Taiwan, Vietnam, Nigeria, and Mexico, among others [5]. HS is an annual plant famous for producing edible red calyxes [5]. The main uses of HS calyxes are culinary, as a source of pigments for cosmetics and food applications, and medicinal in folk medicine to treat many ailments [6]. In general, the therapeutic effects of HS have been associated with the presence of bioactive and functional components such as phenolic acids, flavonoids, anthocyanins, organic acids, and dietary fiber [3].

Several studies in vitro, in silico, and in vivo (often murine models) have demonstrated the pharmacological properties and potential human health benefits of HS consumption [7,8,9]. On the other hand, evaluating the physiological effect of HS in clinical studies is most challenging [10]. In this context, diverse clinical trials have been conducted around the world to demonstrate the ethnopharmacological efficacy of HS for improving human health status in the management of insulin resistance and diabetes mellitus type 2 [11,12], nephropathy [13,14], iron-deficient anemia [15], xerostomic symptoms [16], hypertension [17] and thirst perception in hypertensive patients [18], dyslipidemia [19], liver steatosis [20], overweight/obesity [21], and cardiovascular diseases [22]. Therefore, this narrative review summarizes and discusses the physiological effects and health benefits of HS calyxes reported in clinical trials.

## 2. Traditional Uses and Health Importance of *Hibiscus sabdariffa* Calyxes

Dried HS calyxes are commercially available worldwide and can be used in non-medicinal and medicinal applications [4]. Among non-medicinal uses, especially for food applications, fresh or dried HS calyxes are widely used to make hot or cold drinks, tea, jellies, jams, sauces, wines, syrups, ice cream, and chutneys [5]. Calyxes also add natural food colorants and flavoring to herb teas and bakery products; moreover, calyxes can be roasted and used as a coffee substitute [4,23].

Regarding the medicinal uses of HS, many people rely on herbals as medicinal plants to treat various illnesses in diverse cultures worldwide [4]. In this context, decoction, infusion, or maceration of HS leaves and calyxes have been used as an antimicrobial, antiparasitic, antioxidant, laxative, antispasmodic, diuretic, hepatoprotective, antianemic, anti-inflammatory, analgesic, antitussive, choleretic, antipyretic, hypotensive, cardioprotective, and neuroprotective agent [3,9,23,24,25,26]. Moreover, it is used to treat inebriation [27]. Most of the HS preparations are homemade; however, in some countries, they are commercially available [28]. In a questionnaire-guided survey conducted among students and staff (1238 subjects) of the University of Ibadan in Nigeria, Showande et al. [28] demonstrated that most people (96.9%) had used an HS beverage as a relaxant (29.2%), for blood pressure control (24.3%), for weight reduction (10.7%), for the management of diabetes (11.5%), for infertility treatment (9.7%), and to cure liver diseases (6.7%) [28]. Furthermore, the consumption of HS drinks does not negatively interfere with the pharmacokinetics of acetaminophen [29]. On the other hand, natural supplements/nutraceutical products such as pills and tablets are also commercially available [13].

**Figure 1:**
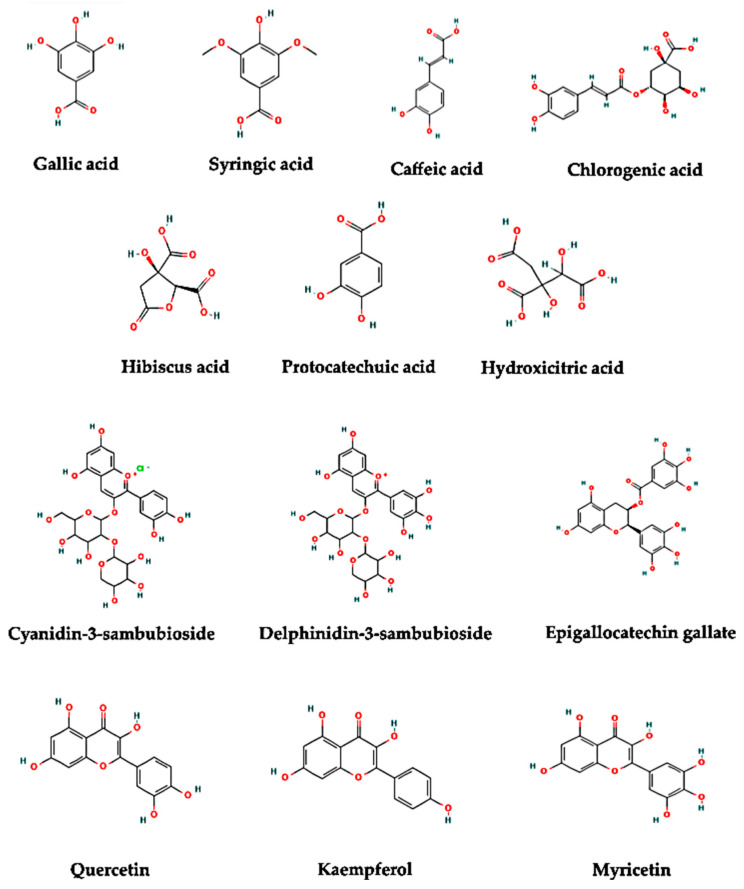
Chemical structures of bioactive compounds identified in *Hibiscus sabdariffa* calyxes. Structures obtained from PubChem database, National Center for Biotechnology Information (gallic acid CID: 370, syringic acid CID: 689043, caffeic acid CID: 689043, chlorogenic acid CID: 1794427, hibiscus acid CID: 6481826, protocatechuic acid CID: 72, hydroxycitric acid CID: 123908, cyanidin-3-sambubioside CID: 3084569, delphinidin-3-sambubioside CID: 74977035, epigallocatechin gallate CID: 65064, quercetin CID: 5280343, kaempferol CID: 5280863, myricetin CID: 5281672).

In this context, the interest in research on HS health benefits has significantly increased globally due to the potential of medicinal uses to prevent or control NCDs [8,30,31,32]. Nonetheless, most biological properties of HS are attributed to the presence of diverse bioactive compounds/secondary metabolites such as phenolic acids, flavonoids, anthocyanins, and organic acids [33,34] (Figure 1), as well as to their bioaccessibility in the gastrointestinal tract [35] and bioconversion during colonic fermentation [36]. Table 1 lists some phytochemical screening of HS calyxes.

## 3. Biological Activities of *Hibiscus sabdariffa* Calyxes with In Vitro and In Vivo Models

**Figure 2:**
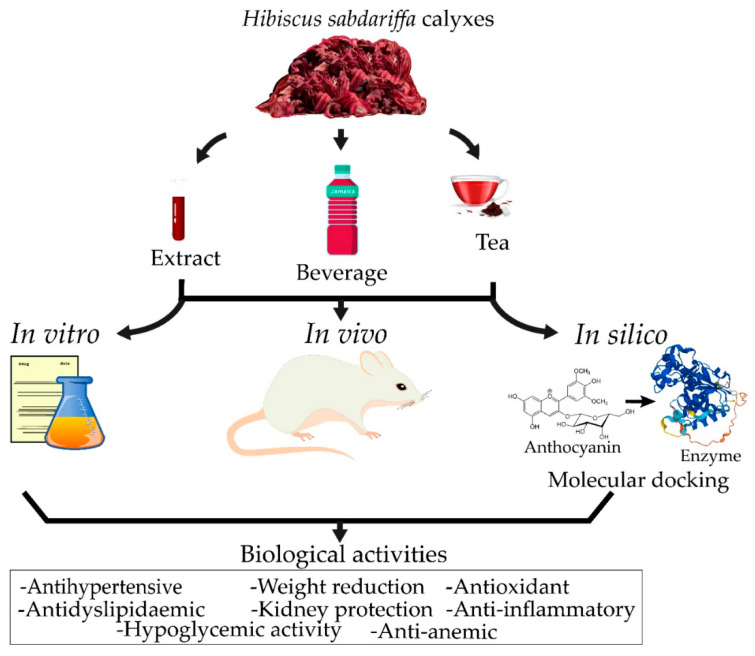
Biological activities of Hibiscus sabdariffa by in vitro, in vivo, or in silico tests.

Several in vitro and in vivo (often murine modelled) biological activities of HS have been reported in literature, including antioxidant [35,55], antihypertensive [34], antidiabetic [56], vasorelaxant [57], cardioprotective [58], antibacterial [59], antiviral [60], antiproliferative [61], cytotoxic [59], neuroprotective [62], sedative [63], antianxiety [64], antidepressant [64], hepatoprotective [65], antihyperinsulinemic [66], anti-obesity [67], anti-inflammatory [68], antianemic [69], and anti-ulcer activities [70], among others, associated with the presence of bioactive compounds, as shown in Figure 2.

Table 2 lists some in vitro and in vivo studies on the biological and beneficial health effects of HS calyxes.

In general, different in vitro assays have been used to evaluate the potential biological properties of HS (Table 2). The antioxidant properties of HS extract have been investigated by 2,2-diphenyl-1-picrylhydrazyl (DPPH^•^) and 2,2′-Azinobis-(3-ethylbenzothiazoline-6-sulfonic acid) (ABTS^+^) radical scavenging, ferric reducing antioxidant power (FRAP) [35], nitric oxide radical scavenging [75], and Fenton reaction assays [56], while the estimation of lipid peroxidation have been evaluated by thiobarbituric acid reactive substances [65] and, moreover, by enzymatic activities such as superoxide dismutase, glutathione peroxidase, glutathione S-transferase, glutathione reductase, and catalase [76,77]. The antioxidant properties of HS extracts have been associated with the phenolic acids, flavonoids, and anthocyanins and their ability to neutralize free radicals, which depends on their structure, including the number and position of the hydroxyl groups [25,35].

The antihypertensive effects of HS have been evaluated by angiotensin-converting enzyme (ACE) inhibitory assay, where the anthocyanin compounds can inhibit the ACE activity by mode of competitive action [34]. Moreover, the antihypertensive activity of HS extract was also carried out on rat aorta, exhibiting a vasorelaxant effect [57]. Regarding the antidiabetic potential of HS, α-amylase and α-glucosidase inhibitory assays have been used to evaluate bioactivity [56]. Adedayo et al. [56] showed that an HS calyx aqueous extract can inhibit enzymes involved in carbohydrate digestion, attributed to the presence of phenolic compounds; however, this effect was in a concentration-dependent response. On the other hand, the cardioprotective effect of HS calyx aqueous extract has been evaluated on doxorubicin-induced cytotoxicity in H9c2 cells by Hosseini et al. [58], who demonstrated that the extract significantly decreased cell apoptosis after 24 h of exposure in a dose-dependent manner and was associated with the ability of HS extract to reduce oxidative stress.

The antibacterial properties of methanolic extract of HS calyxes have been evaluated by the disk diffusion method against various Gram-negative and Gram-positive bacteria, including *Staphylococcus aureus*, *Bacillus stearothermophilus*, *Micrococcus luteus*, *Serratia mascences*, *Clostridium sporogenes*, *Escherichia coli*, *Klebsiella pneumoniae*, *Bacillus cereus*, *Pseudomonas fluorescence and Listeria monocytogenes* [59,60]. Moreover, the antifungal activity was evaluated against *Candida albicans*, *C. glabrata*, *C. guilliermondii, C. krusei*, *C. parapsilosis*, *C. tropicalis*, *Aspergillus parasiticus*, and *Aspergillus flavus* by the minimum inhibitory concentration test [26]. According to the authors, the antimicrobial properties of HS calyx extract are attributed to the presence of phenolic compounds. On the other hand, Joshi et al. [60] demonstrated that aqueous decoction of HS calyxes exhibited antiviral properties (in a standard plaque assay) against hepatitis A, feline calicivirus, and murine norovirus in a dose-dependent response, where the antiviral activity of HS calyx extract was attributed to the synergistic effect of polyphenols and organic acids.

Additionally, the potential anticancer properties of HS calyx extracts have been evaluated using a murine melanoma cell line (B16-F1), human umbilical vein endothelial cells (HUVECs) [8], and a human breast cancer cell line (MCF-7) [61]. The HS aqueous extract exhibited selective cytotoxicity against the MCF-7 cell line [61], while methanolic extract inhibited melanoma cell growth, migration, and tube formation in a dose-dependent response [8]. These results were attributed to the apoptotic properties of various phytochemicals in the extract, mediated by down-regulation of P13/Akt and Ras/MAPK pathways [8,61].

Regarding in vivo studies, the biological activities of HS have been carried out in murine models using healthy or chronic non-communicable disease-induced animals (Table 2). The neuroprotective properties of HS calyx aqueous extracts have been evaluated in ischemic brain injury-induced adult male Wistar rats [62] and cypermethrin-induced oxidative stress male mouse *Mus musculus* [71]. According to the authors, HS extracts at 500 mg/kg body weight (bw) showed protective effects against neuronal damage. This was associated with the antioxidant properties of phytochemicals and their ability to activate endogenous antioxidant defense systems in the brain [71]. Furthermore, it has been reported that HS extract (100 to 400 mg/kg bw) exhibited sedative properties (similar to diazepam) in apomorphine-induced rats in a dose-dependent manner. These results suggested that the extract contains phytochemicals able to interfere with the dopaminergic neurotransmission system, similar to antipsychotic agents [63]. Gulsheen and Sharma [64] informed that gossypetin (a hexahydroxyflavone extracted from HS calyxes) exhibited antianxiety (at 5 mg/kg bw) and antidepressant (at 20 mg/kg) properties in rats exposed to elevated plus maze and forced swim tests. Moreover, in streptozotocin-induced diabetic mice, the ethyl acetate fraction (rich in quercetin compounds) from HS calyxes significantly improved the cholinergic system and hyperphosphorylation tau signaling [54].

The hepatoprotective properties of HS calyxes aqueous extract have been investigated in Wistar rats injected with 2,4-dinitrophenylhydrazine; nonetheless, at doses of 100 to 200 mg/kg bw, HS extract showed hepatoprotective effects, inhibiting the toxicity of DNPH and normalizing the concentration of the T-BIL and D-BIL in a dose-dependent response and similar to that observed using commercial drugs. These effects were attributed to gossypetin, quercetin, sabdaretin, hibiscetin, delphinidin 3-*O*-sambubioside, and cyanidin 3-*O*-sambubioside and their antioxidant properties [65]. Similar trends were reported in a carbon tetrachloride-induced [78] and acetate-induced hepatotoxicity [79] in rats after HS administration at doses of 0.25 mg/kg bw and 50 mg/kg bw, respectively.

The anti-diabetic activity of HS calyxes has been studied in alloxan-induced [72] and streptozotocin (STZ)-induced [80] diabetic rats. In general, aqueous extracts showed hypoglycemic and antioxidant effects after 21 days of administration in a dose-dependent manner [72], while hydro-alcoholic extract reduced blood glucose at 300 mg/kg bw [80]. Moreover, it has been reported that daily supplementation with 100 mg/kg for 28 days significantly improved the liver morphology in STZ-induced diabetic rats [81]. Furthermore, the administration of a polyphenol-rich extract from HS (100 mg/kg for 28 days) ameliorated oxidative stress damage in the heart of diabetic rats [32]. Bunbupha et al. [66] demonstrated that aqueous extract from HS calyxes exhibited anti-hyperinsulinemic properties (at doses of 50 to 200 mg/kg bw) in high fructose diet-induced insulin-resistant rats, and their effects are similar to metformin (an oral anti-diabetic drug) at 100 mg/kg bw. These anti-diabetic effects of HS are associated with the antioxidant properties of its phytochemicals [32,66].

Additionally, HS calyxes’ anti-obesity properties have been reported in rats under high-fat, high-fructose diets [67]; moreover, aqueous extract from HS dried calyxes exhibited hypocholesterolemic properties in hypercholesterolemic rats at doses of 500 and 1000 mg/kg bw daily for six weeks [74]. On the other hand, in healthy rats, aqueous extract of HS (200 mg/kg bw) reduced total cholesterol levels, increasing high-density lipoprotein (HDL) content [82]. Furthermore, the methanolic calyx extract of HS (100 to 800 mg/kg bw) significantly decreased serum cholesterol and increased the liver marker enzymes (alkaline phosphatase (ALP), alanine aminotransferase (ALT), and aspartate aminotransferase(AST)) in a dose-dependent response compared to the control group [83]. These effects were attributed to the abundant antioxidant phytochemicals in HS extracts [82,83,84]. Additionally, the aqueous extract from HS calyx improved hematological parameters in healthy rats, which may be used to manage anemia [69,85].

The antihypertensive properties of aqueous extracts from HS calyxes have been investigated in salt hypertensive-induced rats [73,86]. The extract attenuated the development of hypertension, possibly associated with the high potassium (K^+^) content, stimulating sodium pump and opening potassium channels and promoting the hyperpolarization of the endothelial cells and the antioxidant properties of HS phytochemicals [73,86]. Moreover, the aqueous extract from HS calyxes significantly reduced blood pressure in a rat two-kidney-one-clip model of hypertension [87]. Rodríguez-Fierros et al. [30] reported that aqueous extract of HS calyxes (2 g/L) improved renal function in a metabolic syndrome rat model by reducing oxidative stress due to the presence of compounds able to promote an increase in the non-enzymatic and enzymatic antioxidant systems.

The anti-inflammatory activity of methanolic extracts from HS calyxes has been investigated in paw edema-induced [68] and physical exercise overtrained [88] rats. Onuka et al. [68] demonstrated that the extracts (100 to 400 mg/kg bw) reduced the paw size edema in less time than aspirin (200 mg/kg bw); however, the effect was in a dose-dependent response. These results were attributed to the down-regulation of the expression of pro-inflammatory mediators promoted by polyphenolic compounds. Bayani et al. [88] reported that the HS extracts (at 500 mg/kg bw) exhibited potent anti-inflammatory properties in overtrained rats and prevented spatial memory impairments, associated with the ability of bioactive compounds to maintain the ratio of IL-1β/IL-1ra levels in plasma and the hippocampus.

The gastroprotective properties of aqueous extracts from HS calyxes have been investigated in indomethacin-induced gastric ulcer rats [70]. Huseini et al. [70] demonstrated that HS extract (at 800 mg/kg bw) significantly reduced the gastric ulcer index (GUI of 8.2) compared to the control group (GUI of 22.9). This effect may be associated with the antioxidant properties of anthocyanins, as demonstrated in ethanol-induced gastric lesion rats [89].

In general, most of the in vivo studies that involve animals are carried out in murine models; however, other animals such as rabbits [90,91,92,93], hamsters [94], cats [95], and guinea pigs [96] have been used to evaluate the significant pharmacological effects of HS calyxes. According to the evidence, extracts from HS calyxes have significant pharmaceutical effects in animal and in vitro models, supporting and providing the scientific basis for the use of HS in folk medicine. Some clinical studies have been carried out to conclusively validate the physiological and beneficial effects of HS calyxes in human health, as discussed below.

## 4. Biological Activities of *Hibiscus sabdariffa* Calyxes in Clinical Trials

**Figure 3:**
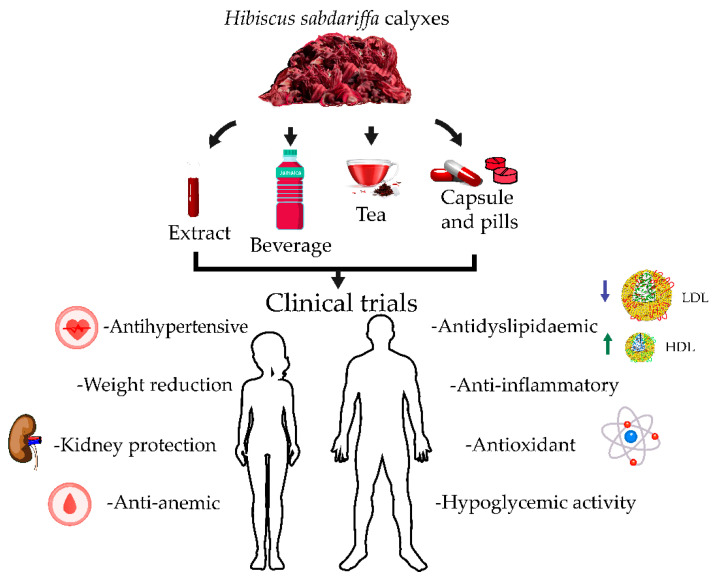
Beneficial effects of *Hibiscus sabdariffa* in humans.

HS calyxes are widely used in non-pharmacological therapy to prevent or control diverse chronic non-communicable diseases [97] associated with their antihypertensive, anti-dyslipidemic, hypoglycemic, antianemic, nephroprotective, antioxidant, anti-xerostomic, and anti-inflammatory properties and body fat mass reduction effects (Figure 3).

### 4.1. Antihypertensive Activity

Hypertension and its complications represent a public health concern globally [98]. The long-term force of blood against the artery wall is high enough that it causes health problems, increasing arterial resistance and reducing the capacitance of venous systems [99]. Hypertension is classified according to systolic and diastolic blood pressure values as pre-hypertense (130–139/85–89 mmHg), grade I (140–159/90–99 mmHg), and grade II (>160/>100 mmHg) [84]. It is treated using pharmacological drugs to avoid disease progression and prevent complications; however, this may produce undesirable side effects [100]. In this context, HS has been used as a natural therapy to control or prevent hypertension [12,17,18,39,51,98,99,100,101,102,103,104,105,106,107,108,109,110,111,112,113,114,115,116,117,118], as shown in Table 3.

In an observational study conducted with one male hypertensive subject (180/120 mmHg, aged 33 years) without pharmacological treatment, Obu [101] demonstrated that the consumption of one single dose of HS tea (two tea bags in 500 mL of boiling water) reduced systolic (from 180 to 150 mmHg) and diastolic (from 120 to 100 mmHg) blood pressure, and these effects were associated with the vasorelaxant properties of HS. Similarly, in an intervention study, the two-times daily consumption of HS tea (2 g of HS in 240 mL of boiling water) reduced systolic (from 115 to 107 mmHg) and diastolic (from 73.38 to 67.19 mmHg) blood pressure of healthy subjects (females between 20 to 35 years) after 48 days of intervention [102].

In a randomized study conducted with 50 mild to moderate hypertensive subjects (25 subjects per group aged 35 to 60 years), Nwachukwu et al. [18] reported that the oral administration of aqueous infusion from HS calyxes (20 g/L of boiling water, corresponding to 10.04 mg of anthocyanin content) significantly reduced systolic (from 150 to 135 mmHg), diastolic (from 100 to 87 mmHg), and mean arterial (from 118 to 105 mmHg) blood pressure and thirst perception (reduction of serum Na^+^ from 140 to 132 mmol/L) in hypertensive patients after 28 days of intervention. According to the authors, the control of hypertension by HS consumption may be related to its angiotensin-converting enzyme (ACE) inhibitory effect and serum sodium balance. Additionally, the effectiveness of HS calyx infusion (20 g in 1000 water, corresponding to 150 mg/kg) in mild to moderate hypertensive subjects (90 subjects, 55 males and 35 females aged 40 to 58 years) was evaluated through a randomized study by Nwachukwu et al. [103], who demonstrated that after 28 days of intervention, HS infusion reduced blood pressure in a dose-dependent response, where a decrease of 11% and 12% in systolic and diastolic blood pressure was observed in subjects with a once-daily dose, while in twice-daily dose decreased blood pressure by 6.9% and 7.4%, respectively. Moreover, the daily consumption of HS did not promote adverse effects in participants.

Jalalyazdi et al. [100] reported, based on a randomized controlled study conducted with 46 hypertensive subjects (130–139/80–89 mmHg) without medication (23 subjects per group aged 18 to 70 years), that with the daily consumption of two cups of HS tea, one in the morning and the other at night (each cup with one tea bag containing 1.25 g of HS powder) for 30 days, subjects exhibited a reduction in systolic (7.43 mmHg) and diastolic (6.70 mmHg) blood pressure compared to those of the baseline (134.61/84.87 mmHg). These results were attributed to the antihypertensive properties of phytochemicals of HS calyxes, and it was mentioned that the effect might come along with dietary and lifestyle modification. Furthermore, it has been reported that the consumption of HS drinks impacts systolic and diastolic blood pressure positively, as demonstrated in a randomized, controlled, cross-over study by Usman et al. [104] in healthy young adult men and women.

Furthermore, the effectiveness of HS infusion (10 g of HS calyx in 500 mL of water, containing 9.6 mg of anthocyanins) to reduce blood pressure has been investigated in a randomized and controlled study conducted with mild to moderate hypertensive patients aged 30 to 80 years by Herrera et al. [105]. They reported that the daily consumption of HS infusion before breakfast for 28 days reduced systolic (from 139.05 to 123.73 mmHg) and diastolic (from 90.81 to 79.52 mmHg) blood pressure, similarly to what was observed in the group treated with captopril at 25 mg twice a day. Moreover, HS infusion exhibited an effectiveness of 78.95% compared to the 84.38% effectiveness of captopril to reduce blood pressure at normal levels; nonetheless, 100% of participants tolerated HS infusion without any adverse effect. According to the authors, the antihypertensive properties of HS products are attributed to the anthocyanin content and their ACE-I and ACE-II inhibitory activities and vasodilator effects [105,106].

The clinical efficiency of HS capsules to control hypertension has been tested by Seck et al. [98] in a randomized controlled study. It was conducted with 83 adults from 25 to 85 years with non-complicated hypertension (SBP of 140 to 175 mmHg and DBP of 90 to 110 mmHg), including 42 subjects in the experimental group and 41 subjects treated with ramipril. They found that after 28 days of daily consumption of HS capsules (320 mg HS powder twice a day), subjects exhibited a reduction in systolic (11.2 mmHg) and diastolic (6 mmHg) blood pressure similar to those of ramipril at 5 mg/day (16 and 5 mmHg, respectively). Moreover, at the end of the intervention, 21% of patients treated with HS and 39% of the subjects in the ramipril group normalized their blood pressure. The antihypertensive effect of HS was related to its diuretic properties through anti-aldosterone activity and its vasodilator effect through ACE inhibition by some phytochemicals. Furthermore, HS capsules were well tolerated, and no significant adverse effects were reported.

In a randomized controlled multicentric study conducted with hypertensive patients aged 40 to 80 years, Bourqui et al. [51] evaluated the antihypertensive properties of HS tea (10 g of calyx/day) or HS capsules (2 capsules of 375 mg per day) compared to the captopril (2 tablets of 50 mg per day) on hypertensive patients (HS capsules, 51 subjects; HS tea, 38 subjects; and captopril, 36 subjects). They demonstrated that after six months of daily consumption of HS products (tea or capsules), patients exhibited a decreased systolic and diastolic blood pressure, where the best results were observed in the HS tea group in a time-dependent response; moreover, HS products showed higher effectiveness (75%) than captopril (65%) to reduce blood pressure to target values (<140/90 mmHg). Most patients exhibited good tolerance to HS products in this study, and only a few cases showed minor adverse effects.

Newly diagnosed (but untreated), mild to moderate hypertensive subjects (25 subjects per group aged 31 to 70 years) were evaluated in a randomized placebo-controlled study after 28 days of daily oral consumption of HS infusion (20 g/L of boiling water) [107]. In this study, HS infusion exhibited higher antihypertensive properties (effectiveness of 76%) than hydrochlorothiazide (HCTZ, 25 mg daily, 60% of effectiveness) without affecting electrolyte balance (Na^+^, K^+^, and Cl^−^) compared to the HCTZ group, where a reduction of K^+^ and Cl^−^ was observed. The therapeutic effect of HS may be associated with a decrease in serum Na^+^ levels and K^+^ and Cl^−^ balance [107].

Additionally, in a sequentially randomized controlled study conducted with 60 mild hypertensive patients with more than a 5 year history of diabetes (ranging from 42 to 63 years), Mozaffari-Khosravi et al. [108] reported that daily consumption of HS tea (2 g-sachets in 240 mL of boiling water, waiting for 20 to 30 min prior to drinking the tea) between main meals (one in the morning and the other in the afternoon) significantly reduced systolic (from 134 to 112 mmHg) and diastolic (from 81.6 to 80.5 mmHg) blood pressure and pulse pressure (from 52 to 34.5 mmHg) after 30 days of intervention. Lately, Mozaffari et al. [109] evaluated the effect of HS tea (3 g sachets in 150 mL of 60–70 °C water, waiting for 15 to 30 min prior to drinking the tea) on the blood pressure of subjects with type 2 diabetes mellitus (patients aged 30 to 60 years with a 5-year history of diabetes) through a randomized clinical study. They showed that the daily consumption of HS tea for 28 days, three times a day (2 h after each meal), significantly decreased systolic (from 119 to 114 mmHg) and diastolic (from 79.4 to 74.5 mmHg) blood pressure, exhibiting a blood pressure-lowering effectiveness of 43% at the end of intervention compared to the baseline of mildly hypertensive subjects with diabetes. The main effect of HS tea was associated with the release of nitric oxide from the endothelium of vessels, preventing the penetration of calcium to vascular smooth muscle cells by the HS phytochemicals; moreover, the authors suggested that consuming HS tea two to three times a day positively influenced blood pressure of diabetic subjects [108,109].

In a quasi-experimental study (with a non-equivalent control group), Ritonga et al. [110] showed that HS extract (10 g of powder brewed with 200 mL of hot water at 90 °C) exerted a reinforced effect of antihypertensive drugs. They found that consuming HS extract (after 3 h) pills reduced the systolic and diastolic blood pressure of postpartum mothers with a history of pre-eclampsia. According to the authors, bioactive compounds such as flavonoids in HS may stimulate or activate endothelium-driven relaxing factors, promoting vasodilatation and preventing ACE-II formation. Harmili et al. [111] investigated the effectiveness of HS tea for reducing blood pressure in hypertensive patients (≥140/90 mmHg, aged 22 to 55 without medication) in a quasi-experimental study with a pre-test and post-test non-equivalent control group (35 patients, 17 in the intervention group). They found that after seven days of daily consumption of HS tea (2 g of HS powder in 150 of boiling water), there was significantly reduced systolic (148.88 to 136.24 mmHg) and diastolic (97.76 to 86.18 mmHg) blood pressure, associated with the vasodilator properties of HS phytochemicals. Moreover, HS tea (2 g HS tea bag in 150 mL of boiling water) consumed twice a day after meals reduced systolic (162.60 to 146.25 mmHg) and diastolic (88.75 to 85.62 mmHg) blood pressure in older women (>60 years), as reported by Yusni and Meutia [112], in a quasi-experimental pre- and post-test control group design; these effects were attributed to the ACE inhibitory properties of the phytochemicals of HS.

Al-Shafei and El-Gendy [17], in a non-randomized quasi-experimental study, reported that the regular consumption of HS infusion (4 standard cups of 250 mL with 2 g HS calyx for 28 days) decreased the pulse pressure (up to 22%) and heart rate (70 to 58 beat/min) in a time-dependent response in moderate essential hypertensive subjects compared to the baseline and normotensive subjects (25 male and 25 female per group aged 45 to 55 years). On the other hand, stopping HS intake elevated the pulse pressure (up to 22.6%) and heart rate (up to 11.4%) of hypertensive subjects, returning to the pre-treatment blood pressure values. Similarly, Haji-Faraji and Tarkhani [113], in a sequential randomized controlled study conducted with 54 moderate hypertensive patients aged 51 ± 10 years (31 subjects in the experimental group), reported that after 15 days of daily consumption of HS tea (two spoonfuls of blended HS tea in 240 mL of boiling water), systolic and diastolic blood pressure significantly decreased (11.2% and 10.7%, respectively) compared to the control group; however, when stopping HS tea consumption, blood pressure returned to the pre-treatment baseline values. Therefore, the regular consumption of HS may progressively reduce or control arterial pressure and heart rate without causing hypotension, possibly by reducing left ventricular pressure overload, which could be related to the ability of HS bioactive compounds to mediate acetylcholine-like and histamine-like mechanisms, promoting vasorelaxant and vasodilator effects associated with the inhibition of Ca^+^-influx into vascular muscle cells via an endothelium-derived nitric oxide-cyclic guanosine monophosphate-relaxant pathway [17].

In a randomized, double-blind, and placebo-controlled study conducted on healthy subjects (aged 18 to 35 years), Kafeshani et al. [114] reported that after 42 days of daily consumption of HS tea (450 mg, containing 250 mg of anthocyanins), significantly reduced systolic but not diastolic blood pressure was observed compared to the placebo group. These effects were attributed to the release of nitric oxide from the endothelium vessels, preventing calcium penetration to vascular smooth muscle cells. Herrera-Arellano et al. [106] investigated the clinical effects of a standardized HS decoction (one enveloped dissolved in 240 mL, containing 250 mg of total anthocyanins) on hypertensive subjects (either sex, aged 25 to 61 years) through a randomized, double-blind, lisinopril-controlled study. After 28 days of daily consumption once a day, subjects exhibited a decrease in blood pressure from 146.48/97.77 to 129.89/85.96 mmHg without significant adverse effects; however, the therapeutic effects of HS were lower than lisinopril at 10 mg (effectiveness 65% vs. 82%). These effects were attributed to the ACE-inhibitory effects of anthocyanins. Moreover, HS extract (150 mg/kg/day for 28 days) reduces plasma aldosterone (32.06%) in newly diagnosed but untreated hypertensive subjects (aged 31 to 70 years), similar to that observed in the lisinopril group (30.01%), as demonstrated in a double-blind controlled randomized study by Nwachukwu et al. [115]. In this study, 76% of subjects treated with HS extracts normalized blood pressure levels, while lisinopril achieved a normalization rate of 65%; moreover, no adverse effects of HS consumption were reported. In this context, HS extracts may reduce hypertension by inhibitory actions on the renin-angiotensin-aldosterone system, such as AT_1_ receptor blocking and Mg^2+^-mediated aldosterone inhibition, associated with the bioactivity of anthocyanins.

In a randomized, double-blind, placebo-controlled study conducted with 65 non-smoking prehypertensive and mildly hypertensive subjects (age 30 to 70), McKay et al. [39] demonstrated that the daily consumption of HS tea (3 cup/day, 3.75 g of HS in 720 mL/day) effectively lowered systolic (129 to 122 mmHg), diastolic (78.9 to 75.8 mmHg), and mean arterial (95.7 to 91.3 mmHg) blood pressure in pre-and mildly hypertensive patients compared to the baseline in a time-dependent response. They showed that after 48 days of intervention, patients’ systolic, diastolic, and mean arterial pressure lowered by 5.5%, 4.0%, and 4.7%; moreover, these effects were higher in patients with higher baseline systolic pressure. According to the authors, the observed blood pressure-lowering effect was associated with the total phenolic (21.85 mg/240 mL) and flavonoid (10.75 mg/240 mL) content of HS tea, particularly by 3-sambubioside and cyanidin-3-sambubioside, which can exert vasorelaxant and ACE inhibitory effects.

Elkafrawy et al. [97] evaluated the antihypertensive efficacy of herbal capsules containing HS and *Olea Europea* (HS-OE, containing 300/200 mg of standardized extracts, respectively) in a phase II, randomized, double-blind, a captopril-controlled study conducted with grade 1 hypertensive subjects (SBP: 140–159 mmHg; DBP: 90–99 mmHg) aged 25 to 60 years. They reported that after 56 days of daily consumption of HS-OE capsules, blood pressure was reduced by up to 15.4/9.6 mmHg, comparable results to captopril at 25 mg (16.4/9.9 mmHg). Moreover, 74% of patients achieved the target blood pressure (<140/90 mmHg) after the intervention, and no adverse effects were reported. These health benefits were associated with the ACE-inhibitory effects of phytochemicals.

Recently, in a pilot comparative intervention, Al-Anbaki et al. [116] evaluated the effect of daily consumption of decocted HS calyx (10 g poured in 500 mL of boiling water) as an alternative treatment for uncontrolled hypertensive patients (≥140/≥90 mmHg). They found that after 42 days of intervention, most of the patients (61.8%) reached the target blood pressure < 140/90 mmHg, associated with the content of anthocyanins (36 mg/10 g of HS calyx) and hibiscus acid (2130 mg/10 g of HS calyx) in the HS powder and their ACE inhibitory and vasorelaxant properties. Similar trends were previously reported in a multicentric pilot clinical study in uncontrolled hypertensive patients (≥140/90 mmHg) after 28 days of daily consumption of HS decoction (10 g poured in 500 mL of boiling water), where 38% of participants quickly reached the target blood pressure. The rest decreased 10 mmHg in the systolic/diastolic blood pressure values. Nonetheless, positive effects on blood pressure were observed after the first week of intervention [99]. According to the authors, HS can help treat hypertension, with or without medication [99,116].

Additionally, it has been reported that a polyphenol-rich HS drink (7.5 g of HS calyx powder in 250 mL, corresponding to 311 mg of gallic acid and 150 mg of anthocyanins) decreased postprandial systolic and diastolic blood pressure in subjects with a cardiovascular risk of 1 to 10% (aged 47 to 49 years) after 4 h of consumption relative to baseline in a randomized, controlled, single-blinded, acute, and cross-over study. These effects were associated with the phenolic compounds (gallic, 4-*O*-methylgallic, and 3-*O*-methylgallic acids) of the HS infusion [12].

Conversely, in a randomized, double-blind placebo-controlled study including 35 participants (17 subjects in the placebo group, aged 42 to 53 years) with metabolic syndrome, it has been reported that the consumption of HS capsules (500 mg of prepared standardized powder containing 6 mg/g of anthocyanins) once daily with meals for 28 days reduced systolic but not diastolic blood pressure [117]. Likewise, Elawad-Ahmed et al. [118] evaluated the effect of HS drink consumption (1.25 g of HS in 300 mL of cold/hot water twice a day) on high blood pressure among hypertensive adult patients in a prospective cohort study including 19 healthy subjects. They demonstrated that after four weeks of intervention, no significant effects were observed in the evaluated parameters and mentioned that the intrinsic characteristic of the population might be a decisive factor.

Additionally, the effectiveness of a phenolic-rich extract of HS combined with *Lippia citriodora* (HS-LC) extract was investigated for reducing blood pressure in a randomized, double-blind, placebo-controlled study conducted with 80 pre-hypertensive or type 1 hypertensive subjects (≥120/80 mmHg) without pharmacological treatment (age 18 to 65 years) by Marhuenda et al. [119], who showed that the daily oral administration of one capsule containing 500 mg of HS-LC extract (325 mg of LC and 175 mg of HS) reduced systolic blood pressure in a time-dependent response, but diastolic blood pressure remained stable after 84 days of intervention. These effects were attributed to the bioactive compounds (type and content) of the plant extracts (anthocyanins and phenylpropanoids); however, they mentioned that the effectiveness of HS for reducing blood pressure is mainly associated with the chemical structure of the bioactive compounds, as well as their bioavailability and bioaccessibility. This information can develop targeted interventions with higher doses, longer duration, and larger sample sizes.

In general, HS’s main antihypertensive action modes are associated with its diuretic, vasodilatation, Ca^+^ influx, and ACE-inhibitory properties and its ability to block AT1 receptors [107]. According to this data, HS could be a natural, available, easy to prepare, and low-cost alternative to control blood pressure in mild to moderate hypertensive subjects without adverse effects.

### 4.2. Anti-Dyslipidemia Activity

Dyslipidemia is considered one of the most critical risk factors for coronary heart diseases and other health complications [19]. It is characterized by an imbalance of lipids mainly due to elevated lipids (cholesterol and triglycerides) and reduced high-density lipoprotein (HDL) levels in the blood, and it is commonly treated with pharmacological drugs [120,121]. The lipid-lowering effects of calyx extracts, capsules, tea, and beverages from HS have been investigated in recent years [9,19,20,22,33,102,117,120,121,122,123,124,125,126], as shown in Table 4.

In healthy subjects without modification in their dietary habits, Tazoho et al. [121] demonstrated that HS tea consumption twice a day (500 mL in the morning and 500 mL in the afternoon, containing 35 g of HS powder/L) for 14 days effectively reduced the total cholesterol (from 134 to 101.7 mg/dL) and low-density lipoprotein (LDL, from 84.70 to 37.97 mg/dL) levels with an increase in HDL (from 32.05 to 42.14 mg/dL) levels. These health effects were attributed to HS’s soluble dietary fiber and phytochemicals.

In an intervention study including 63 healthy female volunteers, the two-time daily consumption of one cup of HS tea (2 g of HS in 240 mL of boiling water) for 48 days inhibited the oxidized low-density lipoprotein levels and downregulated CD36 gene expression [22]. However, HS tea did not change the serum lipid levels (LDL, triglycerides, and total cholesterol) [102].

Additionally, in a randomized controlled study conducted with 43 polygenic dyslipidemia subjects (37 women and 6 men 30 to 60 years old divided into two groups) without using lipid-lowering drugs, Hajifaraji et al. [19] reported that the daily consumption of two cups of HS tea (150 g of HS powder per month) has a positive effect on lipid profile, it decreased total cholesterol (from 246.4 to 223.1 mg/dL) and LDL (from 165.2 to 149 mg/dL) compared to the baseline after 48 days of intervention; moreover, LDL levels slightly decreased from 43.8 to 40.1 mg/dL. These results were attributed to the phytochemical content of HS and its antioxidant properties and the soluble dietary content; however, the authors mentioned that some subjects did not completely adhere to the proposed methodology, which may explain the low effectiveness of HS tea in lowering blood lipids.

The cholesterol-lowering potential of HS calyx extracts was investigated in a randomized crossover study conducted with 42 volunteers (divided into 3 groups and 14 subjects by group, men and women with an average age of 42 years) with elevated cholesterol levels (175 to 327 mg/dL). Participants orally administered one (group 1), two (group 2), or three (group 3) capsules containing 500 mg of HS calyx extract three times daily after meals for 28 days. In general, the oral administration of HS capsules reduced serum cholesterol in a dose-dependent response, with better results in participants who intook two capsules three times daily, reducing total serum cholesterol by 11 to 15% after 28 days of intake. These effects were associated with the anthocyanin (20 mg), flavonoid (10 mg), and polyphenol (14 mg) content of the capsules [33]. Likewise, in a randomized clinical study, it has been reported that the daily consumption of HS capsules (containing 450 mg of HS extract) for 12 weeks effectively lowered total cholesterol (213 to 209 mg/dL), triglycerides (172.32 to 154.47 mg/dL), and serum fatty acids (0.81 to 0.64 U/min per mg of protein) in obese subjects (BMI ≥27, aged 18 to 65), which was associated with the polyphenols of HS, including anthocyanins (2.5%), flavonoids (1.43%), and phenolic acids (1.7%); however, HS capsules did not promote changes in HDL and LDL levels [20].

Sabzghabaee et al. [120], in a triple-blind randomized placebo-controlled study, evaluating the lipid-lowering effect of HS calyx powder (6 g of HS powder per day in divided doses) in 72 obese young subjects (BMI of 25) aged 12 to 18 years with diagnosed dyslipidemia. This study demonstrated that the daily consumption of HS for 28 days significantly reduced serum cholesterol (186 to 176 g/dL), triglycerides (146 to 134 mg/dL), and LDL (111 to 103 mg/dL) levels compared to the baseline values and obtained better results than the drug control group (serum cholesterol from 200 to 198.5 mg/L, triglycerides from 166 to 163 mg/dL, LDL from 125 to 124 mg/dL); however, HS powder did not increase HDL (from 45.64 to 43.17 mg/dL) levels. These results were associated with the antioxidant capacity of polyphenols of HS.

Similarly, in a sequential randomized controlled study conducted with 53 patients with type 2 diabetes (men and women aged average 55 ± 8 years), Mozaffari-Khosravi et al. [122] demonstrated that the daily consumption of HS tea (2 g sachets in 240 mL of boiling water) for 30 days (twice a day after main meals) significantly influenced the blood lipid profile, increasing high-density lipoprotein cholesterol (16.7%) levels and decreasing total cholesterol (7.6%), low-density lipoproteins (8%), triglycerides (14.9%), and Apo-B100 (3.4%) levels compared to baseline at the start of the clinical trial. These results were associated with the water-soluble fibers and the antioxidant compounds present in HS tea.

Additionally, in a factorial, randomized, follow-up study, Gurolla-Díaz et al. [9] reported that oral consumption of HS capsules (100 mg of HS extract, corresponding to 1.42 mg/kg) positively influenced the lipid profile of patients (26 volunteers, men, and women aged between 30 to 71 years) with metabolic syndrome after one month of treatment. Moreover, subjects exhibited a reduction in glucose, total cholesterol, and LDL-c levels, while HDL-c increased without changes in urea, creatinine, alanine aminotransferase, and aspartate aminotransferase (but remained within normal ranges) after treatment compared to the control group (diet); moreover, the authors also reported that these effects were significantly improved in subjects treated with HS capsules plus diet. In general, these effects were associated with the anthocyanin content of HS powder, where delphinid- and cyanidin-3-sambubiosides were the most abundant, indicating the potential use of HS powder as an adjunctive therapy to control or prevent the prevalence of metabolic syndrome.

Asgary et al. [117] showed that the consumption of HS capsules (500 mg of prepared standardized powder, containing 6 mg/g of anthocyanins) once daily with meals for 28 days reduced serum triglycerides but not total cholesterol and LDL values in subjects with metabolic syndrome (35 participants, 17 subjects in placebo group aged 42 to 53 years) in a randomized, double-blind placebo-controlled study. Similar trends were reported by Kuriyan et al. [123] in a double-blind, placebo-controlled, and randomized study (16 subjects, 31 male and 26 female aged between 30 and 60 years with serum LDL values ranging from 130 to 190 mg/dL). This study demonstrated no significant blood lipid-lowering effect in apparently healthy subjects after 90 days of oral administration of 500 mg capsules (twice a day) of leaf hydroalcoholic extract of HS. Differences between clinical studies might be associated with the dosage and frequency of HS consumption, sample size, and study duration [123].

In a quasi-experimental, pre-and post-test study containing a control group conducted with older women (8 subjects per group and >60 years), it has been reported that the daily consumption of HS tea (2 g tea bag in 150 mL of boiling water) twice a day after meals for 21 days significantly reduced total cholesterol (242.62 to 196.25 mg/dL), triglycerides (165 to 140.62 mg/dL), LDL (139.38 to 115.62 mg/dL), and HDL (165 to 36.50 mg/dL) levels compared to the baseline, obtaining better results than the control group, which presented an increase in total cholesterol (256.38 to 265 mg/dL), triglycerides (150.75 to 164.88 mg/dL), and LDL (134.25 to 154.62 mg/dL) levels. These effects were attributed to the phytochemicals of HS and their ability to inhibit the synthesis of triacylglycerol [112]. On the other hand, in a quasi-experimental, pre-and post-test study, Diantini et al. [124] reported that the consumption of an HS calyx beverage (200 mL daily for 30 days) did not promote changes in total cholesterol and LDL levels in healthy adult subjects (17 male and 13 female aged 21 to 55); however, an increase in HDL levels was reported. According to the authors, these effects were attributed to the antioxidant properties of anthocyanins, in particular to the planarity of their aromatic rings able to inhibit xanthine oxidase activity.

AL-Jawad et al. [125] investigated the effect of HS consumption (15 g of HS orally as an infusion before breakfast) on the lipid profile of essential hypertensive patients (15 females and nine males, aged 31 to 65 years) through a prospective randomized clinical case control study. They demonstrated that after 28 days of intervention, subjects exhibited a slight reduction in cholesterol (from 45 to 4.3 mg/dL) and LDL (from 2.53 to 2.19 mg/dL) levels followed by an increase in triglyceride (from 1.8 to 2.0 mg/dL) and HDL (from 1.09 to 1.2 mg/dL) levels, which were associated with the phytochemicals of HS.

Additionally, in a randomized clinical trial, Mohagheghi et al. [126] reported that after 15 days of two times daily consumption of HS tea (500 mg of dry calyxes in two glasses of boiling water), hypertensive patients (51 volunteers, 15 men and 36 women aged average 53 ± 11 years) exhibited an increase in HDL-c (from 44 to 46 mg/dL) without changes in triglyceride, cholesterol, serum creatinine, blood urea nitrogen, and sodium and potassium levels compared to baseline. These results were associated with the short-term administration and the evaluated concentration.

According to these results, HS could be a viable alternative to control or reduce blood lipids in patients with polygenic dyslipidemia, type 2 diabetes mellitus, metabolic syndrome, elevated cholesterol levels, and in obese, overweight, and healthy subjects. However, further studies are needed with large-scale and higher doses to validate the blood lipid-lowering properties of HS effectively.

### 4.3. Hypoglycemic Activity

Diabetes mellitus is a metabolic disease characterized by elevated blood glucose levels, where type 2 diabetes (DM2) is the most common form. It occurs when the body does not make enough insulin or resists insulin. Moreover, eventually, high blood glucose levels can damage blood vessels, eyes, heart, and kidneys, among others [127]. HS has been investigated for its hypoglycemic properties [11,12,13,112,121,127,128], as shown in Table 5.

Harrison et al. [128] evaluated the efficacy of HS tea (10 g of powder in 500 mL of boiling water) consumption in controlling post-prandial blood glucose (60 min) levels of one volunteer for six consecutive days. They found that the HS tea intake in connection with a high-carbohydrate breakfast appears to slow the rise in blood glucose, reducing post-prandial hyperglycemia.

In a quasi-experimental and control group study (12 subjects per group and age of 30 to 60 years), Mayarasi et al. [127] informed that HS-stevia tea (5 g of HS powder and 125 mg of stevia sweetener in 250 mL of boiling water) consumption twice a day for 14 days lowered the fast blood glucose levels of prediabetic women (111.25 to 88.58 mg/dL); these effects were associated with the antioxidant properties of HS, reducing oxidative stress and increasing insulin secretion. On the other hand, 120-min postprandial blood glucose levels were not lowered in research subjects [127].

Additionally, Sarbini et al. [11] evaluated the effect of HS capsules (500 mg twice a day) on insulin resistance in patients with DM2 through a randomized clinical trial. They found that after eight weeks with HS intervention, diabetic patients exhibited a reduction in the fasting blood glucose (from 143 to 119 mg/dL), fasting insulin (from 13.45 to 12.68 µIU/mL), and HOMAR-IR (from 3.17 to 1.73) levels, improving insulin resistance compared to the baseline. These effects were associated with bioactive compounds (flavonoids, phenolic acids, and tannins) that may inhibit key enzymes during carbohydrate digestion, inhibiting glucose tolerance due to insulin resistance and decreasing glucose secretion induced by glucagon insulinotropic polypeptide and glucagon-like polypeptide-1. Moreover, there has been a reported tendency for lower postprandial insulin response in healthy subjects 120 min post-consumption of polyphenol-rich HS beverage (7.5 g of HS calyx powder in 250 mL, corresponding to 311 mg of gallic acid and 150 mg of anthocyanins) compared to the control water-drink group, in a randomized, controlled, single-blind, acute, cross-over study [12].

In a quasi-experimental pre-and post-test control group study, Yusni and Meutia [112] demonstrated that after 21 days of two-times daily consumption of HS tea (2 g HS tea bag in 150 mL of boiling water), elderly subjects (>60 years) significantly reduced postprandial glucose (278 to 225 mg/dL) and cortisol (16.90 to 12.78 µL) levels. According to the authors, the antioxidant phytochemicals of HS play a crucial role in inhibiting cortisol secretion, decreasing gluconeogenesis in the liver, and increasing glycogen synthesis in the liver and muscles.

Conversely, Sakhaei et al. [13] found that daily supplementation with HS pills (425 mg of dried extract containing 5.56 mg of anthocyanins) twice daily did not promote changes in fasting blood glucose levels in patients with diabetic nephropathy in a randomized, double-blind, placebo-controlled study. Similarly, Tazoho et al. [121] found that the oral consumption of sweetened HS calyx beverage (500 mL twice a day, containing 35 g of HS powder/L) did not promote changes in blood glucose of healthy subjects from 21 to 32 years; however, this effect was mainly attributed to the sugar (180 g/L) added in the preparation of the HS beverage.

In general, HS can decrease glycemia in healthy, pre-diabetic, and diabetic subjects, mainly due to its phytochemicals and its ability to modulate carbohydrate digestion and improve insulin secretion and/or sensitivity. However, large-scale studies with more extended periods and higher doses are needed to validate the hypoglycemic properties of HS.

### 4.4. Body Fat Mass Reduction

Overweight and obesity are defined as “abnormal or excessive fat accumulation that presents a risk of health”. They are considered global burden diseases [129]. In this context, HS has been used as an anti-obesity agent to reduce obesity, overweight, and fatty liver [20,21,112,130], as shown in Table 6.

In a randomized clinical study conducted with 36 obese subjects (BMI ≥ 27, aged 18–65), Chang et al. [20] reported that the daily consumption of HS capsules (containing 450 mg of HS extract) for 12 weeks contributed to reducing obesity (reduced weight from 88.52 to 87.28 kg) and abdominal fat (37.37 to 36.67%); moreover, improved liver steatosis was observed. The beneficial effects and metabolic regulation were associated with the polyphenols of HS, including anthocyanins (2.5%), flavonoids (1.43%), and phenolic acids (1.7%), which may act by diverse metabolic pathways, including the up-regulation of PPAR-α expression and inhibition of hepatic lipogenesis [20]. Similarly, Yusni and Meutia [112] reported that 21 days of twice-daily consumption (after meals) of HS tea (2 g HS tea bag in 150 mL of boiling water) reduced body weight (pretest 64.25 kg and posttest 63.38 kg) of older women (>60 years, diagnosed with dyslipidemia, diabetes, and hypertension). This was compared to the results observed in the control group (pre-test 62.72 kg and post-test 62.62 kg) in a quasi-experimental pre-and post-test control group study.

Additionally, it has been reported that two months of daily dietary supplementation of 500 mg capsules containing HS and *Lippia citriodora* polyphenol extracts reduced weight and body mass index of obese (BW from 88.23 to 83.5 kg and BMI from 33.92 to 32.06 kg/m^2^, respectively) and overweight (BW from 67.97 to 64.8 kg and BMI from 26.62 to 25.16 kg/m^2^, respectively) subjects of 45 to 55 years under an isocaloric diet in a double-blind, placebo-controlled, and randomized study. These effects were associated with increased adiponectin levels mediated by the PPARγ pathway [21]. Similarly, a reduction in body weight, BMI, and fat mass were reported in a double-blind, placebo-controlled study conducted with 84 sedentary and healthy subjects (age between 18 to 64 years, divided into two groups) after 84 days of daily consumption of capsules containing HS (175 mg) and *L. citriodora* (375 mg). These effects were associated with the sambubioside and their derivates in both herbal extracts [130].

According to these data, HS has the potential to be used as an adjuvant to control or reduce body weight due to its effect on metabolic regulation, improving liver steatosis. However, further studies should be carried out to find the optimum dosage of HS intake and evaluate the bioavailability and pharmacokinetics of HS.

### 4.5. Nephroprotective Activity

Medicinal plants as phytotherapy have been widely used worldwide as an alternative primary to healthcare, including treating urinary and kidney-related diseases [131,132]. Various clinical studies have been conducted to evaluate the nephroprotective properties of HS [13,105,106,125,131,132,133,134], as shown in Table 7.

In patients with diabetic nephropathy, after eight weeks of supplementation with HS pills (425 mg of dried extract containing 5.56 mg of anthocyanins) twice daily, improved renal function and reduced blood urea nitrogen (33.50 to 24.25 mg/dL), blood creatinine (1.37 to 1.09 g/dL), urine creatinine (934.75 to 742 g/dL), and urine albumin (75.40 to 54.08 mg/L) values after HS supplementation were shown, as well as a significant reduction in high-sensitive C-reactive protein (3.12 to 2.52 µmol/L) levels; these effects were attributed to the bioactive compounds of HS and their antioxidant properties (increased from 23 to 34 µmol/L) being able to reduce oxidative stress [13].

Prasongwatana et al. [131] evaluated the uricosuric clinical effects of HS tea (1.5 of HS powder in 150 mL of boiling water twice a day for 15 days) in healthy and former renal stone subjects (aged 36 to 65 years) in a pre-and post-test study. They found that after taking the HS tea, increases in oxalate, citrate, and uric acid excretion levels were observed in both groups; however, in the renal stone subjects, the effect was significantly increased, which could provide long-term effect benefits for hyperuricemia in gout disease. According to Kirdpon et al. [132], HS calyx beverage did not prevent renal stone formation; however, it may exhibit diuretic effects in healthy subjects (36 subjects without renal diseases between 20 to 30 years) in a dose-dependent response. They demonstrated that 24 g/day of HS extract promoted diuretic effects in healthy subjects, while doses of 16 g/day had no diuretic effect. Moreover, after HS beverage consumption, the urine showed decreased uric acid, creatinine, tartrate, citrate, sodium, calcium, potassium, and phosphate but not oxalate.

In a randomized, double-blind, lisinopril-controlled study, Herrera-Arellano et al. [106] reported that after 28 days of daily consumption once a day of HS decoction (one enveloped dissolved in 240 mL, containing 250 mg of total anthocyanins) in hypertensive subjects, the sodium level showed a tendency to decrease (139.09 to 137.35 mEq/L), while potassium level was not modified, similar to what was observed in the lisinopril control group. Similar trends were reported in mild to moderate hypertensive patients aged 30 to 80 years after 30 days of daily consumption of HS infusion (10 g of HS calyx in 500 mL of water, containing 9.6 mg of anthocyanins), where an increase in sodium excretion was observed with a tendency to decrease chlorine levels without modification of potassium excretion [105].

In a prospective randomized clinical-case control study, AL-Jawad et al. [125] reported that the daily consumption of HS (15 g of HS orally as an infusion before breakfast) for 28 days slightly decreased creatinine and uric acid levels in essential hypertensive patients (15 females and 9 males, aged 31 to 65 years), associated with the presence of flavonoids, anthocyanins, glycoside hibiscus, and gossypetin.

A randomized phase three clinical study was conducted with 93 females (aged 23 to 62 years, divided into two groups) with uncomplicated urinary tract infections; Cai et al. [133] reported that oral supplementation with tablets composed of *Hibiscus sabdariffa* and *Boswellia serrata* extracts (*Acidif plus*) twice a day for seven days reduced symptoms related to urinary tract infections and their recurrence, similar to what was observed with antibiotics. These effects were attributed to the sambubiosides and their anti-inflammatory and antimicrobial properties. Additionally, it has been reported that the oral consumption of HS capsules (500 mg) once a day for 30 days does not cause alterations in renal function of apparently healthy volunteers between 18 to 45 years [134]. According to these data, HS could be used as a diuretic agent without affecting the renal function of healthy and hypertensive subjects.

### 4.6. Antianemic Properties

Anemia is mainly caused by iron deficiency in the blood; it is also associated with low and poor dietary iron intake [15]. In recent years, it has been reported that HS tea can be used for people suffering iron deficits [121]. Peter et al. [15] mentioned that HS extract might exert hematopoietic effects and be used as an herbal supplement to control anemia. They found that the daily consumption of HS extract (1500 mL, equivalent to 1246 mg/g of ascorbic acid) for 30 days increased serum ferritin (9.6 µg/L) compared to the baseline. These effects were associated with the presence of ascorbic acid and other organic acids in HS extract. Tazoho et al. [121] reported that the oral administration of HS calyx beverage (500 mL twice a day, containing 35 g of HS powder/L) improved the hematological parameters of healthy subjects (between 21 to 32 years). They found an increase in red blood cells (3.59 to 4.62 × 10^6^/µL), hemoglobin (11.45 to 14.80 g/dL), packed cell volume (22.95 to 31.94%), and serum iron (101.1 to 108.6 µg/dL) associated with the phytochemicals of HS and their ability to stimulate the formation of erythropoietin in the stem cells; nonetheless, no adverse effects were observed in the kidney in studied subjects.

Moreover, after nine days of twice-daily consumption of an HS water drink (500 mL in the morning and 500 mL in the afternoon), significantly increased hemoglobin levels of apparently healthy subjects between 19 to 39 years under their regular food diet habits were reported [135]. On the other hand, the daily consumption of HS capsules (containing 500 mg of HS ethanolic extract) after dinner for 30 days did not promote changes in hematocrit, platelet, and erythrocyte values of healthy men and women; however, no side effects were reported [136]. According to these data, HS could be used to improve iron deficit in humans because it is an important source of iron and ascorbic acid, favoring the absorption of non-heme iron [137]. However, further studies are required to validate this health benefit effectively.

### 4.7. Antioxidant Activity

The antioxidant properties of diverse plant tissues, including HS calyxes, have been investigated in recent years due to their potential applications in human healthcare, mainly in preventing chronic non-communicable diseases [138]. Recently, in an intervention study including 16 healthy females between 20 to 35 years, Al-Malki et al. [102] found that after twice-daily consumption of HS tea (2 g of HS powder in 240 mL of boiling water) for 48 days, an increase in average antioxidant capacity (from 17.04 to 18.07 mM Trolox equivalent) was detected in blood samples; however, these effects were in a subject-dependent response (43.75% of subjects increased while 37.5% decreased). On the other hand, the authors argued that the main limitation of this study was the sample size.

Abubakar et al. [12] reported that 4 h after polyphenol-rich HS drink consumption (7.5 g of HS calyxes powder in 250 mL, corresponding to 311 mg of gallic acid and 150 mg of anthocyanins), significantly increased antioxidant activity of 25 apparently healthy subjects compared to the control water-drink group was observed in a randomized, controlled, single-blind, acute, cross-over study. These effects were attributed to the antioxidant properties of gallic, 4-*O*-methylgallic, and 3-*O*-methylgallic acids.

Additionally, after 42 days of daily consumption of HS tea extract (450 mg/d), Hadi et al. [139] found an increase in total antioxidant activity (from 1.55 to 1.77 mmol/L) in blood samples of male athletes (54 subjects between 18 to 25 years) with a decrease in malondialdehyde (from 2.67 to 2.26 nm/mL) levels compared to the control group in a randomized, double-blind controlled study, demonstrating that the HS tea extract positively influenced the oxidative stress status of humans.

Frank et al. [138] investigated the antioxidant effects of HS calyx aqueous extract in healthy subjects in a randomized, open-label, and two-way crossover study. They informed that a single dose of HS infusion (10 g of HS powder in 200 mL of water) significantly increased ferric reducing antioxidant power and ascorbic acid levels in blood samples. In contrast, malondialdehyde excretion was reduced compared to the control group treated with water. Moreover, 24 h after HS consumption, an increase in urinary hippuric acid excretion was observed, indicating high biotransformation of the polyphenols of HS (probably caused by gut microbiota). These results demonstrated that the consumption of HS enhanced the antioxidant system and reduced oxidative stress levels in humans.

In a prospective and observational study conducted with 17 patients with Marfan syndrome, Soto et al. [140] reported that daily supplementation with HS calyx infusion (20 g/L of boiling water) for 90 days significantly improved the activities of extracellular superoxide dismutase, glutathione peroxidase, glutathione-S-transferase, and total antioxidant capacity and ascorbic acid compared to patients with Marfan syndrome without supplementation. In this context, HS can increase the antioxidant capacity by enzymatic and non-enzymatic systems in the plasma of Marfan syndrome patients.

In general, the intake of antioxidant compounds is essential to maintain low oxidative stress, which could help to prevent some non-communicable diseases. Further, more extensive studies are needed to provide HS-based therapies effectively.

### 4.8. Other Reported Biological Activities

Other biological activities of HS have been investigated in clinical studies, including anti-inflammatory and anti-xerostomic properties [16,40]. A study conducted with ten healthy adult volunteers (five men and five women aged between 23 and 50 years) by Beltran-Debón et al. [40] reported that a single oral dose of aqueous extract of HS calyxes (10 g of dried calyxes in water) decreased the production of monocyte chemoattractant protein-1 (MCP-1), reducing the expression of interleukine-6 and interleukine-8 three hours after administration. According to the authors, bioactive compounds (mainly anthocyanidin-3-glycosides) present in the aqueous extract may prevent inflammatory and chronic diseases due to the inhibition of MCP-1 production.

Xerostomia is characterized by dry mouth mainly caused by radiotherapy and medications in suffering cancer patients, and it may promote other oral diseases [16]. In this context, HS has been investigated as an alternative to prevent or treat xerostomia. In a prospective, randomized, double-blind placebo-controlled study conducted with 60 xerostomic patients (30 subjects in the treatment group), Levrini and Bossi [16] reported that dietary supplementation of carnosine and dried calyxes of HS tablets (three times during six days) significantly improved the dry mouth symptoms in radiotherapy-induced xerostomic patients (age of 53 ± 20 years), increasing saliva production (27%) with a regulating effect in the pH of saliva in comparison with the placebo control group.

## 5. Reported Limitations during the Clinical Evaluation of *Hibiscus sabdariffa*

Despite the reported physiological and health benefits of HS consumption in humans, the clinical trials carried out to validate the ethnopharmacological properties of HS exhibited various limitations, as listed below.

- Lack of control groups [19,99,108,109];
- The use of HS calyx without extraction [107,108,109];
- The presentation and standardization of HS products [51,98];
- Complete or partial phytochemical characterization and quantification of HS products used in the clinical studies [19,107,108];
- Inherent metabolic characteristics of the studied population [138];
- Small sample size [12,21,39,99,117,118,139];
- Short time for intervention [21,39,99,117,118];
- Doses and frequencies of use reported are heterogeneous among studies [40,114,117];
- No control in the use of pharmacological drugs [110];
- No control over the participant’s eating habits [110,141];
- Some effects are reported in healthy subjects [114];
- The use of an adequate experimental design [17,51,98,109];
- Reliance on subjects and close relatives for treatment adherence [107];
- Lack of data related to the bioavailability during gastrointestinal digestion [39];
- Lack of data related to the biotransformation of HS phytochemicals during gastrointestinal digestion and mechanism of action [39];
- Comorbidities in studied subjects [97].

In this context, some challenges are to be achieved for solving these limitations. Moreover, standardized protocols for the clinical trials of HS calyxes are needed, considering studies with large-scale, higher doses and frequencies of use.

## 6. Conclusions and Perspectives

Significant evidence indicated that consuming decoctions, infusions, beverages, and capsules/pills of HS calyxes exert physiological and beneficial health effects in humans, exhibiting antihypertensive, hypoglycemic, lipid-lowering, antianemic, anti-inflammatory, antioxidant, anti-xerostomic, and diuretic properties; moreover, HS could be used as an adjuvant to control or reduce body weight. These health effects have been attributed to the phytochemicals of HS calyxes such as phenolic acids, flavonoids, anthocyanins, and organic acids and their ability to maintain low oxidative stress in the human body, which could help prevent some non-communicable diseases; furthermore, no adverse effects were reported during the clinical evaluation of HS products. However, further studies on a larger scale and with higher doses are needed to effectively validate the bioavailability and pharmacokinetics associated with the therapeutic effects of HS calyx by-product consumption.

## Figures and Tables

**Table 1:** Bioactive compounds identified in *Hibiscus sabdariffa* calyxes.

Bioactive Compounds	Biological Activity	Reference
Cyanidin-3,5-diglucoside	Anti-inflammatory	[37]
Cyanidine-3-sambubioside	Anti-inflammatory Hepatoprotective Antihypertensive	[38,39]
Delphinidin-3-sambubioside	Hepatoprotective	[40,41]
Delphinidin-3-glucoside	Hepatoprotective	[37]
5-O-Caffeoyl-shikimic acid	Antimicrobial	[42,43]
3-Caffeoylquinic acid	Antioxidant	[38,44]
5-Caffeoylquinic acid	Anti-inflammatory	[38,45]
4-Caffeoylquinic acid	Antimicrobial	[38,46]
4-*O*-methylgallic acid	Antihypertensive Antioxidant	[12]
3-*O*-methylgallic acid	Antihypertensive Antioxidant	[12]
Caffeic acid	Anti-inflammatory	[47,48]
Chlorogenic acid	Immune booster	[35,40,42,49]
Chlorogenic acid isomer I	Immune booster	[40,42,49]
Chlorogenic acid isomer II	Immune booster	[40,42,49]
Epigallocatechin gallate	Antihypertensive	[48]
Gallic acid	Antihypertensive Hypoglycemic Antioxidant	[35,39]
Hydroxycitric acid	Hypolipidemic	[36,40,50]
Hibiscus acid	Vasorelaxant	[36,40,51]
Kaempferol 3-*O*-rutinoside	Anti-inflammatory	[40,52]
Kaempferol 3-(*p*-coumaryl glucoside)	Hypolipidemic	[40,42]
Myricetin 3-arabinogalactose	Hypolipidemic	[40,42]
n-Feruloyl Tyramine	Antioxidant	[40,53]
Protocatechuic acid	Antimicrobial	[43,48]
Quercetin	Antianxiety Antidepressant	[42,54]
Quercitin-3-glucoside	Antianxiety	[42]
Quercetin-3-rutinoside	Antidepressant	[38,42]
Quercetin-3-sambubioside	Antianxiety	[42]
Syringic acid	Antioxidant	[35]

**Table 2:** Biological activities of *Hibiscus sabdariffa* calyxes by in vitro tests and animal models.

Biological Activity	Type of Extract	Concentration/Dose	Model Assay	Main Results	Ref.
In vitro					
Antioxidant	Water-methanol-acetone	NI	DPPH^•^ and ABTS^+^ scavenging and FRAP	Extracts exhibited antioxidant properties	[35]
	Decoction and cold infusions	NI	Briggs–Rauscher oscillating reaction	Beverages exhibited antioxidant properties	[55]
Antihypertensive	Anthocyanin-rich fraction	84.5 µg/mL	ACE inhibitory assay	Fraction exhibited ACE inhibitory activity in a dose-dependent response	[34]
Vasorelaxant	Hexane-ethyl acetate-methanol	0.01 to 2 mg/mL	Rat aorta tissue	Fractions showed vasorelaxant effects by inhibition of Ca^+^ influx	[57]
Antidiabetic	Aqueous	IC_50_ of 25.2 and 187 µg/mL, respectively	α-amylase and α-glucosidase inhibition assay	The extract exhibited higher α-amylase inhibitory activity than α-glucosidase	[56]
Cardioprotective	Aqueous	7 to 500 µg/mL	Doxorubicin-induced cytotoxicity in rat heart-derived myoblast H9c2 cardiac myocyte cells	The extract exhibited a protective effect on cardiomyocytes, increasing cell viability and decreasing cell apoptosis	[58]
Antibacterial	Aqueous-methanolic	20 mg/L	*Staphylococcus aureus*, *Escherichia coli*, *Klebsiellapneumoniae*, and *Bacillus cereus*	The extract exhibited potent antimicrobial activity against Gram-negative bacteria	[59]
Antiviral	Aqueous decoction	100 mg/mL	Feline calicivirus, murine norovirus, and hepatitis A virus	Extracts showed antiviral activity to undetectable levels	[60]
Antiproliferative	Methanolic	0.2 to 1 mg/mL	Murine melanoma cell line (B16-F1) and human umbilical vein endothelial cells	Extract inhibits melanoma cell growth, migration, and tube formation in a dose-dependent response	[8]
Cytotoxic	Aqueous	0.05 to 0.5 mg/mL	Human breast cancer (MCF-7) cell line	The extract showed selective cytotoxic activity in a dose-dependent manner	[61]
In vivo					
Neuroprotective	Aqueous decoction	500 mg/kg bw for 24 days before BCCAO	Ischemic brain injury-induced adult male Wistar rats	The extract showed protective effects against neuronal damage induced by BCCAO	[62]
	Ethanolic	200 to 500 mg/kg bw	Cypermethrin-induced oxidative stress in mice *Mus musculus*	The extract showed protective effects against toxicity induced by cypermethrin	[71]
Sedative	Maceration in hot water	100 to 400 mg/kg bw	Apomorphine-induced stereotypic behavior test using Swiss albino mice	HSE significantly reduced the exploratory behavior in mice, similar to diazepam	[63]
Antianxiety	Ethyl acetate fractions	5 to 30 mg/kg bw	Elevated plus-maze rat model	HS fraction reduced anxiety at low concentrations	[64]
Antidepressant	Ethyl acetate fractions	5 to 30 mg/kg bw	Porsolt’s forced swim test in rats	HS fraction exhibited antidepressant properties at doses of 20 mg/kg	[64]
Hepatoprotective	Aqueous extract	100 to 200 mg/kg bw	Wistar rats injected with 2,4-dinitrophenylhydrazine (DNPH)	The extract inhibited the toxicity of DNPH in a dose-dependent response similar to the control drug	[65]
Cardioprotective	Polyphenol-rich extract	100 mg/kg bw	Hyperglycemia-induced cardiac oxidative stress rats	The extract ameliorated oxidative stress damage in diabetic heart	[32]
Antihyperinsulinemic	Aqueous	50 to 200 mg/kg bw	High fructose diet-induced insulin resistance rats	The extract showed a similar effect to metformin, an oral antidiabetic drug	[66]
Antidiabetic	Aqueous	30 mg/mL	Alloxan-induced diabetic rat	The extract showed hypoglycemic and antioxidant effects	[72]
Anti-obesity	HS by-products	10 g/100 g HF/HFr diet	HF/HFr-induced rats	HS by-products reduced adipocyte hypertrophy, insulin resistance, and hepatic steatosis	[67]
Antihypertensive	Aqueous	6 mg/mL bw	Salt hypertensive-induced rats	The extract attenuated the development of salt-induced hypertension	[73]
Anti-inflammatory	Methanolic	100 to 400 mg/kg bw	Paw edema-induced rat	The extract significantly reduced the paw size edema in a dose-dependent manner in less time than aspirin	[68]
Hypolipidemic	Aqueous	500 to 1000 mg/kg bw	Hypercholesterolemic rats	The extract exhibited hypolipidemic effects in a dose-dependent response	[74]
Renal function improvement	Aqueous	2 g/L	Metabolic syndrome-induced rats	The extract decreased oxidative stress and promoted normal renal function	[30]
Anti-ulcer	Aqueous	100 to 800 mg/kg bw	Indomethacin-induced gastric ulcer rats	The extract exhibited a protective effect against induced gastric ulcer	[70]
Antianemic	Aqueous	200 mg/kg bw	Healthy rats	The extract improved hematological parameters	[69]

NI: no information; BW: body weight; DPPH^•^: 2,2-diphenyl-1-picrylhydrazyl radical; ABTS^+^: 2,2′-Azinobis-(3-ethylbenzothiazoline-6-sulfonic acid); FRAP: ferric reducing antioxidant power; BCCAO: bilateral common carotid artery occlusion; HF/HFr: high-fat high fructose diet.

**Table 3:** Effects of HS consumption on arterial blood pressure parameters.

Country	Sex		Age (years)	Dose	Frequency/Days of Intervention	Sample Size		Design Study	Notes about Participants	Main Results	Ref.
	M	F				Exp.	CT				
Infusion/Decoction/Tea											
Ghana	1	0	33	2 bags *Nyarkotey Tea*	1 day	1		O	HS was consumed 30 min before meals three times.	HS lowers blood pressure	[101]
Saudi Arabia	0	16	25–35	2 g	6 weeks	16		NI	Female participants	HS significantly decreased systolic and diastolic BP in addition to lowering the OxLDL levels of participants	[102]
Nigeria	NI	NI	35–60	150 mg/Kg	4 weeks	25	25	R	Moderate hypertension	Serum Na^+^ decreased significantly in the HS group	[18]
Nigeria	NI	NI	31–70	150 mg/Kg	4 weeks	25	25	R	Participants with newly diagnosed, untreated mild to moderate hypertension	HS obtained greater therapeutic efficacy and duration of antihypertensive action without causing electrolyte imbalance	[103]
Iran	25	21	49.83 ± 3.38	1.25 g	1 month	23	23	R, C	Subjects with stage 1 hypertension	HS significantly decreased systolic and diastolic blood pressure	[100]
Iran	8	45	55.37 ± 8.6 (HS)	2 g	1 month	27	26	R, C	Patients with diabetes and mild hypertension	Ingestion of HS significantly decreased systolic and diastolic BP compared to baseline	[108,109]
Iran	NI	NI	55.5 ± 10.1	Two tablespoons/40 mL water	15 days	31	23	R, C	Patients with moderated essential hypertension	Systolic and diastolic blood pressure decreased significantly when compared to the control group	[113]
Mexico	NI	NI	30–80	10 g	4 weeks	53	37	R, C, DBT	Subjects with hypertension, without drug treatment	Both groups showed significant reductions in blood pressure, with no difference between them	[105]
Indonesia	0	30	NI	10 g	2 days	15	15	Q	Postpartum mothers on antihypertensive drugs	Ingestion of HS showed significant effects on lowering systolic and diastolic blood pressure.	[110]
Indonesia	NI	NI	22–25	2 g	7 days	17	18	Q	Subjects with hypertension	HS reduces blood pressure in pre/post-test measurements and is also compared to the control group	[111]
Indonesia	0	18	>60	2 g twice a day	21 days	9	9	Q, CT	Subjects with metabolic syndrome	HS reduced blood pressure in elderly with hypertension, lowered cortisol, and increased NO	[112]
Egypt	NI	NI	50 ± 5	8 g	8 weeks	50	50	Q, No-R	Patients with moderate essential hypertension and the normotensive general population	Group HS with marked reductions in diastolic, systolic, and pulse pressures	[17]
USA	37	28	30–70	3.75 g	6 weeks	35	30	R, DBT, PC	Systolic blood pressure (120–150 mm Hg) diastolic BP (<95 mm Hg), as inclusion criteria	Lowering of systolic blood pressure	[39]
Iraq	41	90	51 ± 10.3 (HS)	15 g	6 weeks	76	45	Pilot comparative intervention	Subjects without CVD, renal, or retinal complications	HS showed the presence of traces of chlorogenic acid, anthocyanins, and a large amount of hibiscus acid that could participate in the antihypertensive activity	[99]
Capsule/Tablet											
Senegal	NI	NI	53.2 ± 14.3	320 mg twice daily	28 days	42	41	P, R, C	Subjects without evidence of cardiovascular, renal, or retinal complications	Reduction in systolic blood pressure. In addition, 21% of the patients treated normalized their blood pressure vs. 39% of the control group	[98]
Egypt	42	72	20–60	1200 mg HS	8 weeks	39 (Low) 36 (High)	39	R, DBT, C, three-arm	The capsules provided to the subjects were based on extracts of HS and *Olea europaea* (OE).	The consumption of HS-OE reduced blood pressure to values comparable to those of captopril	[97]
Iran	NI	NI	18–35	450 mg	6 weeks	18	18	R, DBT	Healthy adult men	Decreased systolic blood pressure with respect to the control group	[114]
Iran	NI	NI	47.66 ± 4.32 (HS)	500 mg	4 weeks	18	170	DBT, C, CT	Adults with metabolic syndrome	HS decreased systolic blood pressure and TGL with respect to control	[117]
Beverage											
Nigeria	25	25	18–27	500 mL (1.4%)	The mean value of the recordings was recorded in triplicate, after 10 min in the supine position and 5 min standing	25	25	R, C, CO	Subjects with recreational activity without a regular exercise regimen	HS decreased systolic blood pressure and rate pressure product (RPP) induced by standing. The effect on SBP was more significant in women and PRP in men	[104]
Mexico	NI	NI	25–61	250 g	4 weeks	86	84	R, C, DBT	Subjects with hypertension stage I or II	HS significantly reduced plasma ACE activity and showed a tendency to reduce the sodium values	[106]
Senegal	NI	NI	20–70	10 g	1, 3, 6 months	51 (tablet) 39 (brew)	37	R, C	The control group and two experimental groups were contrasted	HS in both presentations was as effective as standard treatment during a 6-month follow-up	[51]
Nigeria	55	35	NI	150 mg/Kg	4 weeks	NI	NI	R	Subjects with mild to moderate hypertension	A low dose (150 mg/kg) of HS once daily reduced blood pressure hypertension	[107]
Nigeria	NI	NI	31–70 49.92 ± 3.40 (HS)	150 mg/Kg	4 weeks	26 (HS) 23 (lisinopril)	26	R, DBT	Mild to moderate hypertension, newly diagnosed, but not treated	HS significantly decreased systolic BP	[115]
UK	NI	NI	49 ± 2	7.5 g	4 h	22	22	R, C, SB, CO		Improved vascular function	[12]
Saudi Arabia	NI	NI	NI	1.25 g	4 weeks	19	0	P	The sample ended with 15 subjects	No significant effects of HS use on blood pressure or lipid profile were observed	[118]

HS: *Hibiscus sabdariffa*; M: male; F: female; Exp: experimental group; CT: control group; O: observational; NI: no information; OxLDL: low-density lipoprotein from human plasma, oxidized; R: randomized trial; C: controlled trial; BP: blood pressure; DBT: double-blind trial; controlled trial; Q: quasi-experimental; NO: nitric oxide; PC: placebo control; CVD: cardiovascular disease; OE: *Olea europaea*; P: prospective trial; TGL: triglycerides; RPP: rate pressure product; ACE: angiotensin converting enzyme; CO: cross-over trial; SB: single-blind trial.

**Table 4:** Effects of HS consumption on blood lipids.

Country	Sex		Age (years)	Dose	Frequency/Days of Intervention	Sample Size		Design Study	Notes about Participants	Main Results	Ref.
	M	F				Exp.	CT				
Infusion/Decoction/Tea											
Saudi Arabia	0	16	25–35	2 g	6 weeks	16	0	NI	Female participants	Lowering the OxLDL levels of participants. Anthocyanins were able to inhibit OxLD	[22,102]
Iran	6	37	30–60	2 g	12 weeks	21	22	R, C	Adults with polygenic dyslipidemia	The intervention group showed a significant reduction in total cholesterol and LDL-c	[19]
Iran	35	37	14–21	2 g	NI	36	36	R, P, C	Adolescent population	Serum total cholesterol, LDL-c, and TGL showed a decrease in all cases	[120]
Iran	8	45	55.37 ± 8.6	2 g	1 month	27	26	R, C	Patients with diabetes	HS decreased TGL, CT, LDL-c, and APO-B100 levels; it also increased HDL-c levels	[122]
Iran	NI	NI	50 ± 14	15 mg	30 days	42	42	R	Patients with hypertension were treated for at least 3 months.	No significant differences were found between pre and post-experiment measurements in the groups evaluated	[126]
Iraq	9	15	31–65	15 g	4 weeks	NI	NI	P, R, CC	Hypertension patients	The HS group showed the most significant decrease in blood pressure, cholesterol, and LDL and a decrease in urea nitrogen	[125]
Indonesia	0	18	>60	2 g twice a day	21 days	9	9	Q, CT	Subjects with metabolic syndrome	HS group reduced TC, TG, HDL, and LDL in the elderly with dyslipidemia	[112]
Capsule/Tablet											
Mexico	NI	NI	30–71	100 mg	1 month	51	73	R, P	Metabolic syndrome diagnosed according to NCEP-ATP III criteria	Hypolipidemic and hypotensive effects were reported.	[9]
China	16	26	18–75	500 mg per capsule	4 weeks	NI	NI	R, CO	Volunteers with elevated cholesterol without supplement or medication intake	HS promoted a significant decrease in serum cholesterol levels	[33]
China	21	15	18–65	450 mg	12 weeks	19	17	DBT, R	Obese subjects	HS capsules did not promote changes in HDL and LDL levels	[20]
Iran	NI	NI	47.66 ± 4.32 (HS)	500 mg	4 weeks	18	170	DBT, C, CT	Adults with metabolic syndrome	HS decreased systolic blood pressure and TGL with respect to control	[117]
India	31	26	35–60	1 g	90 days	28	29	R, DBT, PC	Subjects without chronic diseases with 130–190 mg/dl serum LDL values.	The experimental group showed a 10% reduction in triglyceride values, and the placebo group showed no significant change	[123]
Beverage											
Cameroon	32	0	25.38 ± 3.35	35 g	2 weeks	32	0	NI	Subjectswere advised not to consume HS from any other source during the experimental period	Reduced the total cholesterol and LDL-c and increased HDL-c	[121]
Indonesia	17	13	39.33 ± 9.18	200 ml	30 days	NI	NI	NI	Healthy adults	HS significantly lowered blood pressure and increased HDL	[124]

HS: *Hibiscus sabdariffa*; M: male; F: female; Exp: experimental group; CT: control group; NI: no information; OxLDL: low-density lipoprotein from human; R: randomized trial; C: controlled trial; TC: total cholesterol; LDL-c: low-density lipoprotein-cholesterol; P: prospective trial; TGL: triglycerides; APO-B100: apolipoprotein B-100; HDL-c: high-density lipoprotein-cholesterol; CC: clinical case-control; BP: blood pressure; Q: quasi-experimental; CO: cross-over trial; DBT: double-blind trial; PC: placebo control.

**Table 5:** Effects of HS consumption on blood glucose levels.

Country	Sex		Age (years)	Dose	Frequency/Days of Intervention	Sample Size		Design Study	Notes about Participants	Main Results	Ref.
	M	F				Exp.	CT				
Infusion/Decoction/Tea											
Indonesia	NI	NI	30–60	5 g	14 days	98	103	Q	Pre-diabetic women	HS significantly decreased fasting blood glucose levels with no effect on postprandial glucose	[127]
Indonesia	0	18	>60	2 g twice a day	21 days	9	9	Q, CT	Subjects with metabolic syndrome	Postprandial glucose and cortisol levels were significantly reduced in subjects > 60 years of age	[112]
USA	4	4	18–43	10 g	6 days	6	6	NI	One sample was collected per day for 6 days, 60 min after breakfast	HS together with a carbohydrate-rich breakfast appeared to slow the rate of increase in the glucose curve	[128]
Capsule/Tablet											
Indonesia	NI	NI	35–65	1 g	8 weeks	30	30	Exp, DBT	The control group and the HS group presented homogeneous conditions	HS 500 mg 2 times a day can significantly reduce fasting blood glucose and insulin levels	[11]
Beverage											
UK	NI	NI	49 ± 2	7.5 g	4 h	22	22	R, C, SB, CO		After 120 min of HS consumption, there was a tendency to a lower postprandial insulin response than the control group	[12]
Iran	22	38	52 (mean)	425 mg	8 weeks	30	30	R, DBT, PC	Of the participants in the study, 80% used insulin, and 20% used glucose-lowering drugs	HS did not promote changes in fasting blood glucose levels in patients with diabetic nephropathy	[13]
Cameroon	32	0	25.38 ± 3.35	35 g	2 weeks	32	0	NI	The preparation of the beverage granted contained sugar (180 g/L)	HS did not promote changes in blood glucose in healthy subjects aged 21 to 32 years	[121]

NI: no information; HS: *Hibiscus sabdariffa*; M: male; F: female; Exp: experimental group; CT: control group; Q: quasi-experimental; DBT: double-blind trial; C: controlled trial; SB: single-blind trial; CO: cross-over trial; R: randomized trial; PC: placebo control.

**Table 6:** Effects of HS consumption on body weight.

Country	Sex		Age (years)	Dose	Frequency/Days of Intervention	Sample Size		Design Study	Notes about Participants	Main Results	Ref.
	M	F				Exp.	CT				
Infusion/Decoction/Tea											
Indonesia	0	18	>60	2 g twice a day	21 days	9	9	Q, CT	Subjects with metabolic syndrome	HS reduced body weight vs. control group (pretest/posttest)	[112]
Capsule/Tablet											
China	21	15	18–65	450 mg	12 weeks	19	17	DBT, R	Obese subjects	HS reduced obesity and abdominal fat	[20]
Spain	0	55	36–69	250 mg HS mixture with *Lippia citriodora* (LC)	8 weeks	29	26	R, DBT, PC	Healthy women 24–34 kg/m^2^	The LC-HS groups showed a more significant decrease in body weight abdominal circumference, mainly in overweight individuals	[21]
USA	NI	NI	18–64	175 mg HS, 375 mg LC	84 days	42	42	R, PC, DBT	Healthy subjects	LC-HS significantly decreased body weight	[130]

HS: *Hibiscus sabdariffa*; M: male; F: female; Exp: experimental group; CT: control group; Q: quasi-experimental; ***vs:*** versus; DBT: double-blind trial; R: randomized trial; LC: *Lippia citriodora;* PC: placebo control; NI: no information.

**Table 7:** Effects of HS consumption on renal function.

Country	Sex		Age (years)	Dose	Frequency/Days of Intervention	Sample Size		Design Study	Notes about Participants	Main Results	Ref.
	M	F				Exp.	CT				
Infusion/Decoction/Tea											
Mexico	NI	NI	30–80	10 g	4 weeks	53	37	R, C, DBT	Subjects with hypertension, without drug treatment	Increased sodium excretion, a tendency for chlorine levels to decrease, no change in potassium excretion	[105]
Iraq	9	15	31–65	15 g	4 weeks	NI	NI	P, R, CC	Hypertension patients	Decrease in BP and decrease in urea nitrogen	[125]
Thailand	NI	NI	36–65	1.5g	15 days	9	9	NI	Healthy males	Uric acid excretion and clearance increased significantly	[131]
Capsule/Tablet											
Italy	0	93	22–63	100 mg HS, 100 mg *Boswellia serrate*, 400 mg L-methionine	30 days	46	47	R	Treatment combined	In the HS group, 95.7% of patients showed significant clinical improvement compared to women treated with standard antibiotic therapy	[133]
Indonesia	10	10	18–45	500 mg	30 days	20	0	NI	Healthy volunteers	HS does not affect renal function in healthy subjects	[134]
Beverage											
Iran	22	38	52 (mean)	425 mg	8 weeks	30	30	R, DBT, PC	Of the participants in the study, 80% used insulin, and 20% used glucose-lowering drugs	Reduction in blood urea nitrogen, blood creatinine, urine creatinine, and urine albumin values, as well as a significant decrease in high-sensitivity C-reactive protein	[13]
Mexico	NI	NI	25–61	250 g	4 weeks	86	84	R, C, DBT	Subjects with hypertension stage I or II	Showed a tendency to reduce sodium, while the potassium level did not change	[106]
Thailand	36	0	20–30	16 g–24 g/day	2 weeks	36	0	NI	Healthy subjects	Urinary excretion decreases creatinine, uric acid, citrate, tartrate, calcium, sodium, potassium, and phosphate	[132]

Exp: experimental group; SB: single-blind trial; CO: cross-over trial; O: observational; Q: quasi-experimental; HS: *Hibiscus sabdariffa*; M: male; F: female; CT: control group; NI: no information; R: randomized trial; C: controlled trial; DBT: double-blind trial; P: prospective trial; CC: clinical case-control; BP: blood pressure; PC: placebo control.

## Data Availability

Not applicable.
